# Eccentricity pacing and rapid termination of the early Antarctic ice ages

**DOI:** 10.1038/s41467-024-54186-1

**Published:** 2024-12-05

**Authors:** Tim E. van Peer, Diederik Liebrand, Victoria E. Taylor, Swaantje Brzelinski, Iris Wolf, André Bornemann, Oliver Friedrich, Steven M. Bohaty, Chuang Xuan, Peter C. Lippert, Paul A. Wilson

**Affiliations:** 1 University of Southampton, Waterfront Campus, National Oceanography Centre Southampton, Southampton, UK; 2 Department of Earth Sciences, University College London, London, UK; 3 School of Geography, Geology and the Environment, University of Leicester, Leicester, UK; 4 Department of Earth and Environmental Sciences, The University of Manchester, Manchester, UK; 5 Department of Earth Science, Bjerknes Centre for Climate Research, University of Bergen, Bergen, Norway; 6 Institute of Earth Sciences, Ruprecht-Karls-Universität Heidelberg, Heidelberg, Germany; 7 Institute of Geosciences, Goethe-University Frankfurt, Frankfurt, Germany; 8 Federal Institute for Geosciences and Natural Resources, Hannover, Germany; 9 Department of Geology & Geophysics, University of Utah, Salt Lake City, UT USA

**Keywords:** Palaeoclimate, Palaeoceanography

## Abstract

Earth’s obliquity and eccentricity cycles are strongly imprinted on Earth’s climate and widely used to measure geological time. However, the record of these imprints on the oxygen isotope record in deep-sea benthic foraminifera (δ^18^O_b_) shows contradictory signals that violate isotopic principles and cause controversy over climate-ice sheet interactions. Here, we present a δ^18^O_b_ record of high fidelity from International Ocean Drilling Program (IODP) Site U1406 in the northwest Atlantic Ocean. We compare our record to other records for the time interval between 28 and 20 million years ago, when Earth was warmer than today, and only Antarctic ice sheets existed. The imprint of eccentricity on δ^18^O_b_ is remarkably consistent globally whereas the obliquity signal is inconsistent between sites, indicating that eccentricity was the primary pacemaker of land ice volume. The larger eccentricity-paced early Antarctic ice ages were vulnerable to rapid termination. These findings imply that the self-stabilizing hysteresis effects of large land-based early Antarctic ice sheets were strong enough to maintain ice growth despite consecutive insolation-induced polar warming episodes. However, rapid ice age terminations indicate that resistance to melting was weaker than simulated by numerical models and regularly overpowered, sometimes abruptly.

## Abstract (web summary)

High fidelity benthic foraminifera oxygen isotope records reveal that variations in Earth’s orbit are the primary pacemaker of change in early Antarctic ice ages and caused these ice ages to sometimes end abruptly.

## Introduction

**Fig. 1:**
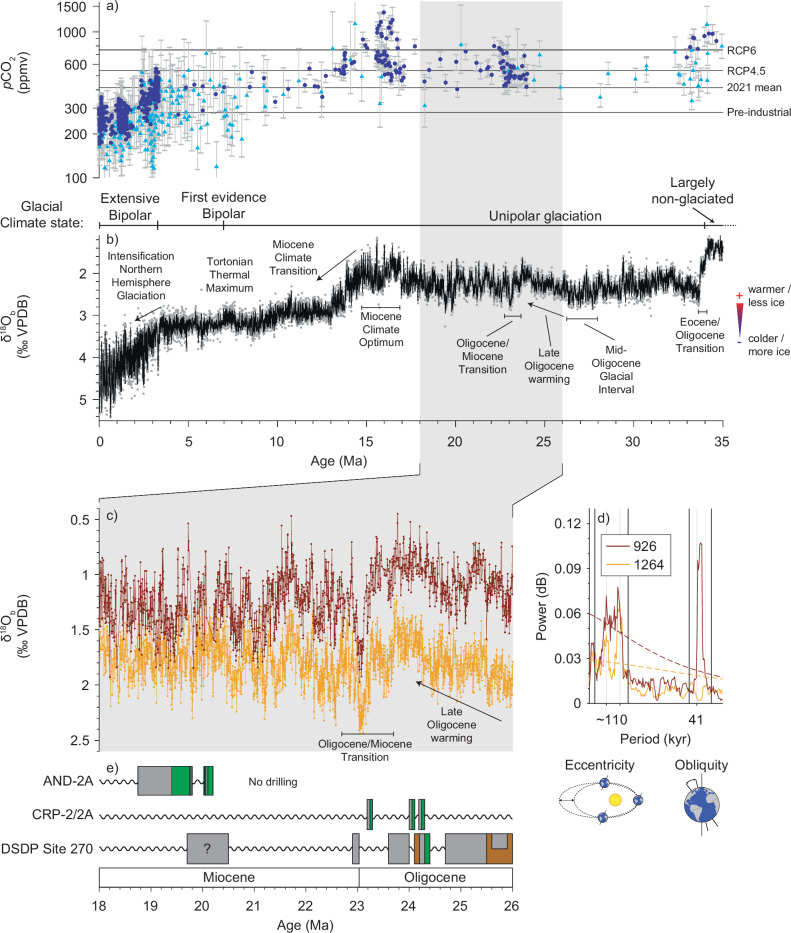
Contrasting indications of the astronomical pacemaker of Antarctic glacial-interglacial cycles during the high CO_2_ mid-Cenozoic unipolar icehouse. **a** atmospheric carbon dioxide (*p*CO_2_) reconstructions with 2σ uncertainties^7^, from phytoplankton (cyan triangles) and boron isotopes (blue circles). **b** marine benthic foraminiferal oxygen isotope (δ^18^O_b_) composite record CENOGRID^5^ with 10-point (black) LOESS smoothing, relevant climate events and glacial states shown. **c** contrasting astronomical pacing of two marine δ^18^O_b_ records (ODP sites 926 and 1264, refs. ^19,29^) 26−18 Ma. **d** power spectra for the data shown in c. Vertical black lines denote bandwidths used throughout this paper. **e** Antarctic (ice-proximal) records AND-2A, CRP-2/2A, and DSDP Site 270 with colours representing simplified lithology from refs. ^18,22,23^: light grey mudstone (often including ice-rafted debris), dark grey sandstone, green diamictite, brown sandstone, and sinusoidal lines disconformities. The question mark in DSDP Site 270 denotes uncertain lithological age. Site locations shown in Figure S1.

Oxygen isotope data from benthic foraminifera (δ^18^O_b_) record changes in Cenozoic climate. The overall pattern of change suggested by pioneering δ^18^O_b_ studies^1^ and subsequent composites of higher resolution work^2–5^ reveals pronounced global warmth during the early Eocene followed by long-term cooling and growth of polar ice sheets. Ice sheet growth was likely conditioned by slowly declining atmospheric carbon dioxide (CO_2_) levels^6,7^ (Fig. 1a) and geologically sudden threshold-breaching jumps in response^8–10^. The Cenozoic increase in Earth’s land ice budget was strongly diachronous between hemispheres. Large ice sheets were first sustained on Antarctica around 34 million years ago (Ma)^11–13^ (Fig. 1b). The transition to bipolar glacial conditions occurred much later^13^. A Greenland Ice Sheet may have developed by 11−7 Ma (refs. ^14,15^), even though isolated glaciers may have existed during the middle Eocene^16^, but it was not until about 2.6 Ma that extensive ice sheets waxed and waned over North America and Eurasia^17^ (Fig. 1b). Thus, for about 30 million years, nearly half of Cenozoic history, Earth’s ice age history was primarily written by the growth and decay of Antarctic ice sheets. This unipolar icehouse climate state (Fig. 1b) presents an opportunity to examine a future-relevant high CO_2_ climate system with Antarctic-only ice sheet variability, but it is understudied compared to the bipolar icehouse climate state, especially the great ice ages of the late Pleistocene.

A mechanistic understanding of Antarctic ice sheet (in)stability must incorporate astronomical forcing. Palaeoclimate records from locations both proximal and distal to Antarctica show unequivocal imprints of astronomical forcing^18–21^. Ice-proximal records reveal direct evidence of glacial advances and retreats over the drill sites that are linked to the Antarctic hinterland, yet the same glacial advances cause hiatuses. Distal δ^18^O_b_ records facilitate long-term continuous evolutions of astronomical pacing of global ice volume and temperature yet could be affected by preservation issues or low sedimentation rates, smoothing out higher-frequency signals. The lack of a coherent picture from Antarctic ice-distal and ice-proximal records—information that should be complementary—is particularly acute for the late Oligocene-to-early Miocene interval (herein referred to as the Oligo-Miocene, ~28−20 Ma). Glacio-marine sedimentary sequences recovered from the Ross Sea (Fig. S1) show unequivocal evidence of multiple episodes of ice sheet advance and retreat during the Oligo-Miocene (Fig. 1)^18,20,22,23^ that were astronomically, mainly obliquity paced^18,24^. A recent review of ice-proximal records from multiple Antarctic locations^25^ highlights a wealth of past climate data (e.g., refs. ^26,27^) that support dynamic oceanic environments related to ice age variability. During short intervals of the Oligo-Miocene, both obliquity (41 thousand years [kyr]) and short eccentricity (110-kyr) cycles have been observed at different locations (e.g., refs. ^18,24,28^) and may thus ultimately drive ice sheet variability in the hinterland of those localities^24,28^.

Records from deep-sea sediments at ice sheet-distal sites complement ice-proximal records by providing the astronomical pacemaker of the globally integrated cryosphere system, rather than individual ice margins or sheets, over prolonged periods of time. Deep-sea sediment records are typically continuous over long periods of time and can therefore pick up differences in ice sheet variability across changes in astronomical configuration. Yet, the astronomical imprint on the pre-Quaternary deep-sea δ^18^O_b_ record is far from clear cut. Published records for the Oligo-Miocene are especially puzzling because they show pronounced spatial and temporal variability both in amplitude and response to astronomical forcing^19,21,29–31^ (as synthesized in Fig. 1). This arrhythmia in the δ^18^O_b_ record (Fig. 1c, d), unless explained by markedly different signal fidelity between sites, presents a major challenge to interpreting palaeoclimate because it contradicts the fundamental principles of oxygen isotope stratigraphy: on timescales longer than the mixing time of the ocean (~1.5 kyr), the ice-volume signal encoded in δ^18^O_b_ must be common to all records of sufficient fidelity^32^. Here, we present a δ^18^O_b_ record of exceptional fidelity from the North Atlantic Ocean (Supplementary Data 1) and use it as a benchmark to address the arrhythmia identified in the published δ^18^O_b_ records and to evaluate the astronomical pacing of the early Antarctic ice ages in synthesis with Antarctic proximal records.

## Results and Discussion

### A δ^18^O_b_ record from Site U1406

At Site U1406, clay-rich nannofossil oozes were recovered from a sediment drift at 3.8-km water depth on the Newfoundland margin in the Northwest Atlantic Ocean hosting well-preserved calcareous microfossils^33^ (Figure S1). Before now, δ^18^O_b_ data for most of the Cenozoic from this climate-sensitive region (the North Atlantic Ocean) were sparse because of large gaps in the geological record attributable to discontinuous coring, hiatuses, and condensation horizons^34,35^. Our δ^18^O_b_ record spans approximately 26.4 to 21.8 Ma across the Oligocene-Miocene Transition, a critical interval to the debate about the sensitivity of the early Antarctic ice sheet to astronomical forcing and the mechanisms controlling the evolution of those ice ages^18–20,31,36^. With a temporal resolution of generally 1–3 kyr, our record is the best-resolved δ^18^O_b_ time series available from anywhere in the world for the Oligo-Miocene interval. Our δ^18^O_b_ record was constructed using exceptionally well-preserved benthic foraminifer *Cibicidoides mundulus* (Methods) from a sequence characterised by rates of sedimentation between 1 and 3 cm kyr^−1^ and placed on an astronomically tuned age model that is independent of our δ^18^O_b_ data (Supplementary Data 2, SI Astronomical Tuning, Data Quality Control).

**Fig. 2:**
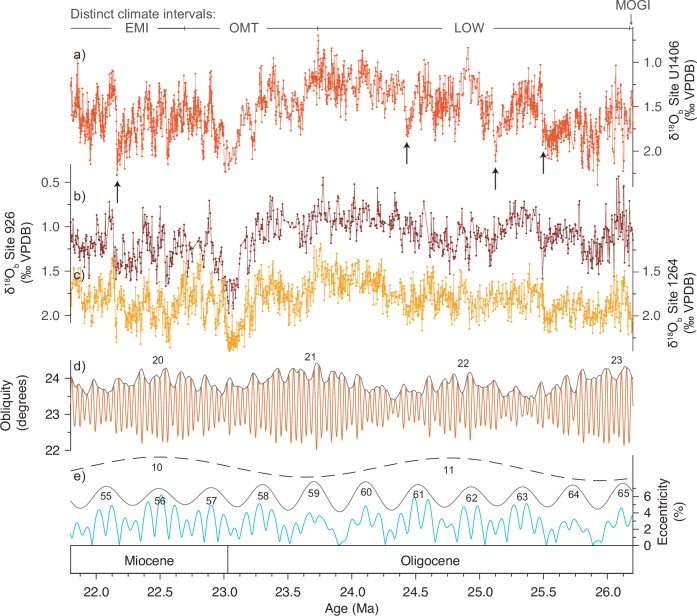
Our δ^18^O_b_ record from IODP Site U1406 compared to the records from sites 926 and 1264 and the astronomical series. **a** δ^18^O_b_ from Site U1406 (bright red, this study). EMI (Early Miocene Interval), OMT (Oligocene-Miocene Transition), LOW (Late Oligocene Warming), and MOGI (Mid-Oligocene Glacial Interval). Arrows indicate abrupt decreases in δ^18^O_b_ at Site U1406. **b** and **c** δ^18^O_b_ at sites 926 (dark red)^29^ and 1264 (yellow)^19^, respectively. **d** and **e** La2004 astronomical solutions^41^ for obliquity and eccentricity, respectively, with obliquity instantaneous amplitude and long-term eccentricity modulations of ~405 kyr and ~2.4 Myr. Site locations shown in Fig. S1.

We recognise three main intervals with distinct climatic imprints in our δ^18^O_b_ record from Site U1406 (Fig. 2a). Our record begins close to the transition between the ‘middle’ Oligocene glacial interval (MOGI, ~28−26.3 Ma), when high δ^18^O_b_ values indicate a cold deep glacial climate state with high-amplitude glacial-interglacial cycles^19^. There follows an interval of inferred long-term deglaciation through the late Oligocene (‘Late Oligocene Warming’ LOW; ~25.7−23.7 Ma) related to warming and/or continental subsidence in West Antarctica^19,37,38^ within which three abrupt, high amplitude (up to ~1 ‰) decreases in δ^18^O_b_ are revealed at ~25.5, ~25.1, and ~24.4 Ma (Fig. 2a, arrows). An interval of high-amplitude variability in δ^18^O_b_ with a pronounced overall transient increase delineates the rapid major changes in glacial state of Antarctica during the Oligocene-Miocene Transition (OMT; ~23.7−22.6 Ma). High-amplitude variability, including a fourth prominent abrupt decrease in δ^18^O_b_ at ~22.1 Ma, is also characteristic of the early Miocene interval (EMI, ~22.6−21.8 Ma). Three of these four events initiated when δ^18^O_b_ was close to peak values for the data set (≥2.1‰ ± 0.17 ‰, Fig. 2). Two of those documented at Site U1406 (at ~25.5 and ~22.1 Ma) occur at the start of maxima in ~405-kyr Eccentricity Cycles 63 and 55 and are also present (although less well resolved) in the δ^18^O_b_ records of sites 926 and 1264 (Fig. 2b, c)^19,29^. The δ^18^O_b_ decreases identified at ~25.1 and ~24.4 Ma are not distinct at sites 926 and 1264 (Fig. 2b, c)^19,29^.

### A strong globally consistent response of δ^18^O_b_ to eccentricity

**Fig. 3:**
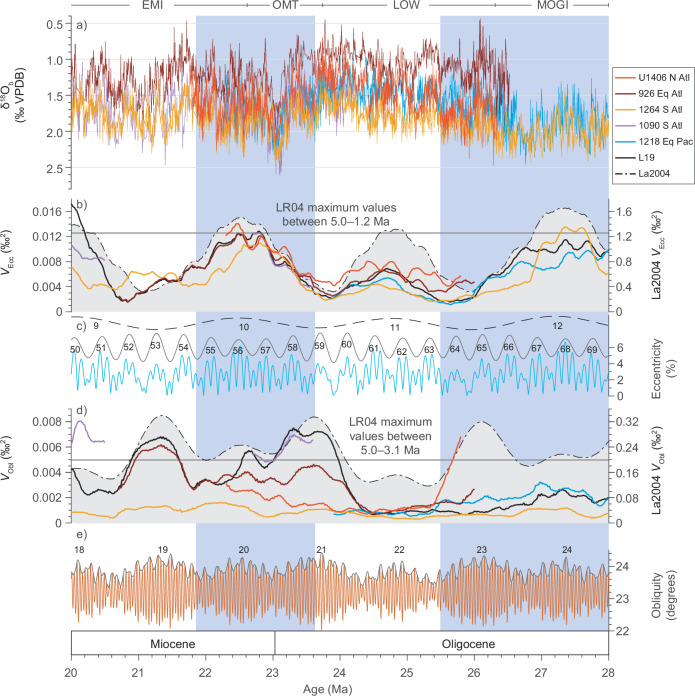
A globally coherent response to eccentricity and inconsistent response to obliquity in the Oligo-Miocene δ^18^O_b_ record. **a** δ^18^O_b_ of ODP/IODP sites U1406 (bright red; presented here), 926 (dark red)^29^, 1090 (purple)^30^, 1218 (blue)^21^, and 1264 (yellow)^19^ during EMI (Early Miocene Interval), OMT (Oligocene-Miocene Transition), LOW (Late Oligocene Warming), and MOGI (Mid-Oligocene Glacial Interval). Blue shading indicates colder intervals (roughly equivalent to the MOGI/LOW transition and OMT interval) with high-amplitude variability in δ^18^O_b_ on frequencies corresponding to eccentricity. **b** eccentricity variance (*V*_Ecc_) of the δ^18^O_b_ records shown in a), the composite δ^18^O_b_ record^20,47^ and the La2004 eccentricity solution^41^ shown in (**c**) with long-term eccentricity modulations of ~405 kyr and ~2.4 Myr offset for clarity. Grey line denotes maximum *V*_Ecc_ values in the 5.0−1.2 Ma interval of the LR04 δ^18^O_b_ stack^39^ (see Fig. S3). **d** obliquity variance (*V*_Obl_) of the δ^18^O_b_ records shown in a), the composite δ^18^O_b_ record^20,47^, and La2004 obliquity solution (La2004, dashed)^41^ shown in (**e**) with amplitude modulation. Grey line denotes maximum *V*_Obl_ values in the 5.0−3.1 Ma interval of the LR04 δ^18^O_b_ stack^39^ (see Fig. S3). Note that the y-axes of *V*_Obl_ and *V*_Ecc_ are scaled differently to allow site-to-site comparisons (see Fig. S2b for plot with identical y-axes). Site locations shown in Fig. S1.

To assess the astronomical forcing of Oligo-Miocene climate, we calculated the obliquity variance (*V*_Obl_) and eccentricity variance (*V*_Ecc_) (Methods) for selected δ^18^O_b_ records and our δ^18^O_b_ record from the North Atlantic (SI Data Quality Control). Both *V*_Obl_ and *V*_Ecc_ show pronounced changes over the Oligo-Miocene study interval (Fig. 3). We highlight two main observations on the temporal and spatial variability in *V*_Ecc_. First, *V*_Ecc_ almost always exceeds *V*_Obl_ in all records (Fig. S2b). The same result is also clearly seen in the Middle-to-Late Pleistocene of the δ^18^O_b_ record LR04^39^ (Fig. S3d, e) when ~100-kyr pacing dominated glacial-interglacial cycles^40^. Second, throughout our Oligo-Miocene study interval, we find that *V*_Ecc_ is remarkably similar among all records and strongly resembles the long-term (~2.4-million years [Myr]) modulation of the La2004 eccentricity solution (Fig. 3b)^41^. The analysed time interval spans most of ~2.4-Myr Eccentricity Cycle 12 through to the first half of Eccentricity Cycle 9 (Fig. 3c). The highest consistently recorded *V*_Ecc_ values of ~0.010−0.014 ‰^2^, indicative of high-amplitude ~110-kyr eccentricity cycles in δ^18^O_b_, occur during ~2.4-Myr Eccentricity Cycles 12 and 10 in the La2004 astronomical series^41^. During ~2.4-Myr Eccentricity Cycle 11, concurrent with the LOW interval, the response of δ^18^O_b_ to eccentricity is modest with *V*_Ecc_ values ranging between ~0.004 and 0.008 ‰^2^ (Fig. 3b). The higher *V*_Ecc_ values during ~2.4-Myr Eccentricity Cycles 12 and 10 (Fig. 3b) are associated with generally higher absolute δ^18^O_b_ values (colder/more ice) during the MOGI, OMT and EMI (Fig. 3a). Thus, our analysis reveals a relationship wherein cooler more deeply glaciated Oligo-Miocene climate states are coupled with a stronger imprint of Earth’s eccentricity than warmer less glaciated climate states. This result is consistent with the one established more recently in the Pleistocene^39^ when ice sheets in the Northern Hemisphere dominated glacial-interglacial climate variability (Fig. S3).

### Site-to-site variability in the response of δ^18^O_b_ to obliquity

In contrast to the globally consistent picture seen in *V*_Ecc_, we see striking site-to-site variability in *V*_Obl_ for most of the studied Oligo-Miocene time interval (Fig. 3d). Before we can consider environmental explanations for this observation, we must first assess processes that may bias δ^18^O_b_ records and artificially introduce site-to-site variability, especially in higher frequency bands (here *V*_Obl_). These include low sedimentation rates, under-sampling, the effects of stratigraphic discontinuities, age models that inadvertently tune power into or out of the obliquity bandwidth, and foraminiferal species and preservation effects on δ^18^O_b_ (summarized below, Supplementary Data 3, and SI Data quality control). The record from Site 1090 is from a mix of genera and species^30^ and it is not sampled at high enough resolution to assess *V*_Obl_ between 22.5−20.5 Ma (Fig. 3b, d, S2f). The record from Site 1218 is also multi-species and is too sparsely resolved to resolve obliquity-paced variability from ~23.2 Ma and younger. At Site 926, the δ^18^O_b_ record was produced using a mixture of *Cibicidoides* species in the interval between ~26.5−25 Ma (ref. ^29^) but uses only *Cibicidoides mundulus* between 25.0−17.9 Ma and is a particularly useful comparison to our record because of its temporal resolution, site location and water depth (see below). The record from Site 1264 is derived from a single species (*Cibicidoides mundulus*) and sampled at high resolution, but, because sedimentation rates are low (often ≤ 1cm kyr^−1^, Fig. 3b, d, S2g), it is likely that the obliquity-paced signal is damped or even removed by sediment mixing (bioturbation).

Our record from Site U1406 provides a benchmark against which to assess Oligo-Miocene variability in *V*_Obl_ because (i) it benefits from a strong astrochronology independent of δ^18^O_b_ (Supplementary Information), (ii) was developed using unusually well-preserved, mono-specific samples of *Cibicidoides mundulus*, and (iii) comes from a site with relatively high sedimentation rates (~1‒3 cm kyr^−1^) (Methods) that is sampled at high resolution (generally 1−3 kyr sample spacing) to resolve obliquity cycles throughout. We draw three major findings by comparing our record to published records from other sites. First, records from all sites, including U1406, show a long-term minimum in *V*_Obl_ within the LOW ~25.4−24.4 Ma (Fig. 3d) that indicates little response to modest obliquity forcing (Fig. 3e). Second, the striking site-to-site variability in *V*_Obl_ that characterizes the older and younger parts of our Oligo-Miocene record (~25.8–25.4 and ~24.4−22.6 Ma) becomes especially pronounced when a strong response to modulated obliquity forcing emerges at some sites, but not others, during minima in *V*_Ecc_ and in the ~2.4 Myr amplitude modulation of eccentricity (Fig. 3b, c). One example occurs close to the MOGI-LOW transition (25.8–25.4 Ma; Fig. 3d) when baseline δ^18^O_b_ values are high and the global imprint of ice volume changes on δ^18^O_b_ was large^19,42^. We attribute the higher *V*_Obl_ seen in our North Atlantic record from Site U1406 to a combination of higher sedimentation rates, higher temporal resolution compared to the other records available (Fig. S2f), and the use of a single species (whereas a mix of species was used at sites 926 and 1218) (Supplementary Data 3). A second example of pronounced site-to-site variability in *V*_Obl_ occurs between the culmination of LOW and the OMT (~24.4−22.6 Ma), when δ^18^O_b_ values and the imprint of ice volume changes on δ^18^O_b_ ranged between the lowest and highest values in the whole of our record (Fig. 3a, d)^19^. Here, sedimentation rates and temporal resolution among the records (Fig. S2f, g) are more similar across sites, but *V*_Obl_ in the North Atlantic Site U1406 is two to four times lower than in the equatorial Atlantic (Site 926) and sub-polar South Atlantic (Site 1090) despite obliquity tunings underpinning the age models at all three sites (Fig. 3d).

The offset in *V*_Obl_ between U1406 and 926 between ~24.4−22.6 Ma is especially significant because the record from Site 926 was also produced using monospecific foraminifera in this interval. Therefore, the inconsistency between these two records in this interval cannot be readily explained by taxonomic biases and instead suggests an environmental control. The most likely explanation for the observed contrasting *V*_Obl_ is lower amplitude changes in bottom water temperature (BWT) at the mid-northern-latitude Site U1406 than at the equatorial Site 926. Consequentially, the influence of the Deep Western Boundary Current (DWBC) on the mid-depth Oligo-Miocene western North Atlantic Ocean may have been substantially weaker than today, because sites U1406 and 926 lie in very similar water depths (~3.8 km and 3.6 km, respectively) and are both bathed today by the same water mass (DWBC). Southern Component Waters (SCW) may have influenced changes in BWT at Site 926 more than at Site U1406, possibly only during obliquity minima as demonstrated by increased corrosiveness^31,36,43^. Before ~24.4 Ma, Antarctic margin cryosphere processes influenced BWT more similarly at both sites. The reduced influence of SCW at Site U1406 from 24.4 Ma onwards is part of a reorganization in ocean circulation, as shown by deep-water flow speed increases in the Southern Ocean in the latest Oligocene^44–46^. Such change in ocean circulation has also been hypothesized to be one of the causes of the major Antarctic glaciation at the Oligocene-Miocene Transition^44^, in addition to a favorable astronomical configuration^29,31,41^ and a decreasing *p*CO_2_ concentration during the late Oligocene^7^.

The culmination of this major Antarctic glaciation at the Oligocene-Miocene Transition was proceeded by high-latitude climate cooling, a change in tectonic setting ^19,37,38^, and an expansion of the Antarctic ice sheet into marine settings^18,20,25,38^. The margins of these ice sheets were, at times, astronomically paced^18^. To establish the sensitivity of these margin to orbital forcing, Levy et al. (ref. ^20^) calculated obliquity sensitivity (*S*_Obl_), a ratio between *V*_Obl_ in δ^18^O_b_ (climate response in the obliquity bandwidth) and *V*_Obl_ in the astronomical solution (climate forcing by obliquity), using a composite δ^18^O_b_ record^47^ for the interval between ~34 and 5 Ma. This revealed an abrupt increase in *S*_Obl_ at ~24.5 Ma (Fig. 3d), which is only apparent in some of the δ^18^O_b_ records (Fig. S2c), but not in ours. We attribute the *S*_Obl_ jump^20^ visible in the δ^18^O_b_ megasplice at ~24.5 Ma to the tie point between the two spliced records (1218 and 1090)^21,30^ with contrasting *S*_Obl_ (Fig. 3, S2c, S4). Our analysis of all δ^18^O_b_ records shows markedly different obliquity-paced changes among sites. Therefore, we suggest that the mechanism(s) that drove high-latitude ice volume and BWT variability in the late Oligocene were less sensitive to changes in obliquity than indicated by a subset of δ^18^O_b_ records and some ice-proximal sedimentary records.

### Eccentricity pacing of the Antarctic Ice Sheet

The site-to-site congruence in the deep-sea δ^18^O_b_ record in the eccentricity bandwidth is unmistakable within the Oligo-Miocene interval (Fig. 3b) and strongly suggests a common cause that is imprinted similarly in all records. We attribute this result to the waxing and waning of ice sheets on Antarctica because the ice volume component in δ^18^O_b_ is globally uniform on astronomical time scales and because ice sheets did not develop extensively in the Northern Hemisphere until the latest Pliocene^17^. Eccentricity pacing of the Oligo-Miocene Antarctic ice sheets is consistent with a record of seawater δ^18^O (δ^18^O_sw_) change for the OMT^48^, which suggests a transient sea-level fall of up to ~50 meters sea-level equivalent (msle) at the Oligocene-Miocene Transition. This is similar to the up to 30−40-msle estimates from inverse modelling of 1-D ice sheets^49^ and backstripping estimates^50^. Our analysis reveals major glacial-interglacial cycles in Antarctic climate and ice sheet size during the late MOGI into early LOW and from peak OMT into EMI (Fig. 2), which were predominantly paced by eccentricity between 26 and 20 Ma. Obliquity pacing of larger volumes of Antarctic ice may have occurred when Earth’s orbit was circular (i.e., no eccentricity)^42^ and occurred regularly much later, e.g., around the Miocene Climate Optimum (18−13 Ma)^20,51^. Throughout this time, ice-proximal evidence shows that eccentricity-modulated precession also still paced volumetric changes in land-based ice^52,53^.

The amplitude of the eccentricity-paced variations in δ^18^O_b_ suggest waxing and waning of a large proportion of the Antarctic ice sheet. We note that absolute δ^18^O_b_ values and ice-proximal sedimentological evidence^22,25^ indicate sufficiently large ice volumes during the OMT climate event that the Antarctic ice sheet transiently expanded over the continental shelf into the marine realm. However, the high-amplitude δ^18^O_b_ variations together with palaeotopographic and erosion rate reconstructions for the entirety of Antarctica in the Oligo-Miocene^54,55^ (see below) emphasise regular waxing and waning of the large land-based ice sheets on Antarctica at other times (not only the OMT climate event). Moreover, strong eccentricity pacing of Oligo-Miocene ice ages, together with strengthened evidence for abrupt decreases in δ^18^O_b_ registering glacial termination events (note arrows in Fig. 2a), suggests ice sheet hysteresis behaviour that was much weaker than currently modelled for Antarctica^56–59^ but strong enough to allow ~110-kyr (not ~20-kyr or 41-kyr) ice age cycles. Further analysis of this problem is merited to understand why the largely land-based early Antarctic ice sheet, despite its pole-centred configuration, shows cyclical behaviour akin to the late Pleistocene Laurentide Ice Sheet (LIS). As for the LIS, the early Antarctic ice sheet clearly underwent sustained growth over successive insolation cycles (Fig. 3). Presumably, the abrupt Antarctic ice age termination events (Fig. 2) were driven by an astronomically paced increase in radiative forcing and lagged glacial bedrock isostatic rebound, but, while the LIS contributed to its own demise by advancing deep into the mid-latitudes^60–62^, the same process is not possible on Antarctica.

The Middle to Late Miocene transition to an obliquity-paced Antarctic ice sheet, perhaps more vulnerable to ocean melting than before^20^, was likely partly caused by the ice sheet itself as it advanced and eroded the Antarctic continental shelf^63^. Antarctic palaeotopographic maps and offshore sediment accumulation rates^54,55^ reveal where the Antarctic ice sheet was most active in the mid to late Cenozoic. Estimated offshore long-term erosion rates point to a dynamic East Antarctic Ice Sheet (EAIS) during the Oligo-Miocene, especially in the low-lying Recovery, Aurora, and Wilkes subglacial basins^54,55^. In contrast, West Antarctic erosion rates appear to have been relatively subdued during the Oligo-Miocene compared to the last ~14 Myr^54^. Smaller ice sheets with marine margins in West Antarctica and on the Antarctic Peninsula should not be discounted^22,64^ and would have contributed to transient changes in sea-level. Large parts of West Antarctica, however, lay above sea level during the late Oligocene and early Miocene^54^ providing nucleation points for ice caps to form^65^. We suggest, therefore, that the congruent behaviour of *V*_Ecc_ seen in globally distributed δ^18^O_b_ records during the Oligo-Miocene was mainly driven by waxing and waning of land-based Antarctic ice sheets and included East Antarctica due to the amplitude of the δ^18^O_b_ changes. This suggestion is consistent with palaeotopographic maps and offshore sediment accumulation rates^54,55^.

The direct effect exerted by Earth’s eccentricity on insolation is too weak to drive major changes in climate directly. As a result, the strong response of the Antarctic climate-cryosphere system on eccentricity time scales must originate from a nonlinear response to eccentricity-modulated precession forcing^41^. Results of a detailed trace element study^48^ of benthic foraminifera from Site 926 across the OMT interval suggest an amplifying mechanism rooted in the carbon cycle and reconstructions of the sensitivity of the Antarctic ice sheet for that interval^66^ are consistent with those of coupled climate-ice sheet model experiments^57^. One mechanism that may explain amplification of eccentricity-paced climate forcing is the destabilization of methane gas hydrates after a sufficient drop in sea level like at the OMT climate event^67^. This negative feedback mechanism could drive a relatively quick glacial termination which is visible in these global δ^18^O_b_ records (e.g., at ~22.1 Ma, Fig. 2). Another mechanism may occur at low latitudes and involves the monsoon-driven modulation of global marine organic carbon burial rates, resulting in eccentricity-paced oscillations in atmospheric CO_2_^68^. Further work is needed to explore the influence of low-latitude processes on astronomically paced changes in polar air temperature during the Cenozoic and to improve our understanding of hysteresis behaviour of the early Antarctic ice sheets.

## Methods

### Isotope geochemistry

Discrete samples of approximately 20 cm^3^ were taken at a sample spacing of 2−4 cm between 54.76 and 155.08 m CCSF-M using the revised splice^69^. Half of these samples (n = 1446; 54.76−107.97 and 146.50−155.08 m CCSF-M) were processed at the University of Southampton's Waterfront Campus, National Oceanography Centre Southampton (UoS-NOCS), and the other half were processed at the University of Leipzig (UoL, n = 467; 108.00−126.85 m CCSF-M), University of Frankfurt (UoF, n = 396; 126.89−145.65 m CCSF-M), and University of Heidelberg (UoH, n = 353; 116.08−145.90 m CCSF-M) using standard methods. Samples were oven-dried (40−50°C), washed over a 63-μm sieve, and oven-dried again. Weights were recorded between every processing step, and the wt.% coarse fraction (>63 μm) was determined for every sample (Supplementary Data 1). To obtain a robust isotopic value for each sample level and minimize isotopic variability attributable to ontogenetic effects, three to eight of the best preserved *Cibicidoides mundulus* specimens were selected from the 125–250 μm sieve fraction and transferred into reaction vials. We used *C. mundulus* because the isotopic offset from seawater equilibrium values is well documented for this epifaunal species^70–72^. Where insufficient *C. mundulus* were present in the 125–250 μm fraction, additional individuals were picked from the 250–355 μm size fraction. Stable oxygen isotope data (Supplementary Data 1) were generated using a Thermo Fisher Scientific MAT 253 mass-spectrometer coupled to a Thermo-Finnigan Kiel IV Carbonate Device in Southampton, Heidelberg, and Leipzig, and coupled to a Gasbench II in Frankfurt. International standards (NBS-18 and NBS-19) and in-house quality control standards were used to calibrate δ^18^O_b_ across laboratories, yielding a reproducibility for δ^18^O_b_ in the range of 0.05−0.08‰ (±1σ) over the 30-month measurement period.

### Site selection and statistical data processing

Published qualitative observations suggested that obliquity pacing was strongly expressed in δ^18^O_b_ records from the western equatorial Atlantic Ocean at Ocean Drilling Program (ODP) Site 926 (Fig. 1c, d)^29,31^ and the eastern sub-Antarctic Atlantic at ODP Site 1090^30^. In contrast, short (~110 kyr) eccentricity was identified as the primary pacemaker of the δ^18^O_b_ record from the eastern subtropical South Atlantic Ocean (Site 1264), where no strong obliquity signals were detected (Fig. 1c, d)^19^. These three sites (ODP sites 926, 1090, and 1264)^19,29,30^ fall along a latitudinal transect from the equatorial to South Atlantic Ocean. A fourth δ^18^O_b_ record from the equatorial Pacific Ocean (Site 1218)^21^ combined a varying mix of both eccentricity and obliquity signals.

To quantify the obliquity and eccentricity pacing, we calculated the obliquity variance (*V*_Obl_) and eccentricity variance (*V*_Ecc_) of all records. First, we removed substantial visual outliers from all δ^18^O_b_ records (*i.e*., from sites 926, 1090, 1218, 1264, and U1406). Next, records were linearly interpolated to 4-kyr, approximately equivalent to the coarsest continuous sampling resolution available, which is more than sufficient to statistically capture the obliquity cycle^73^. Subsequently, the δ^18^O_b_ records were detrended using a high-pass filter preserving frequencies >1 cycle Myr^−1^. We used Astrochron^20,74^ to compute *V*_Obl_ and *V*_Ecc_ as well as obliquity sensitivity (*S*_Obl_) over frequencies of 25 ± 2 cycles Myr^−1^ and 9 ± 3 cycles Myr^−1^ that correspond to the 40-kyr obliquity cycle and the ~110-kyr eccentricity cycle, respectively, across 1-Myr-long sliding windows. We also computed *V*_Obl_ and *V*_Ecc_ of a composite δ^18^O_b_ record^47^ comprising data spliced together from ODP sites 926, 1090, and 1218, from which obliquity sensitivity was also computed by ref. ^20^.

### Carbonate content calibration

Bulk carbonate content strongly covaries with the natural logarithm of calcium over potassium counts, i.e., ln(Ca/K), calculated from XRF core scanning data^69^ (Fig. S5a). ln(Ca/K) was calibrated to coulometric CaCO_3_ concentration using an exponential fit (Fig. S5a). We used both shipboard^33^ and shore-based coulometry data (wt.% CaCO_3_) generated at UoS-NOCS, using a CM5015 coulometer equipped with an AutoMate automated analysis device (Supplementary Data 4). Three samples were selected to evaluate measurement precision: These samples were all run three times, yielding mean wt.% CaCO_3_ contents of 34.9 wt.%, 11.3 wt.%, and 17.8 wt.% with a precision at 1σ of 0.35 wt.%, 0.25 wt.%, and 0.47 wt.%, respectively.

### Phase calculations and wavelet analysis

We obtained coherency and phase estimates between CaCO_3_ content and δ^18^O_b_ (Fig. S5b) on the initial magnetostratigraphic age model^75^ using the SPECTRAN software^76^ and a Parzen window across 33% of the data series. Phase and coherency estimates were calculated in the obliquity bandwidth of 23 ± 2 cycles Myr^−1^. The computational settings for phase and coherency estimates included 2σ error bars and yielded coherency confidence levels of 80% and 95%.

Morlet wavelet analysis^77,78^ were used for time-frequency analysis in both depth and age domain, after linear interpolation to 2 cm (XRF-based CaCO_3_ in depth) and 4 kyr (XRF-based CaCO_3_ and δ^18^O_b_ in age) and detrending using a high-pass filter preserving frequencies >0.04 cycle m^−1^ (preserving cycles with a period <25 m) or >1 cycle Myr^−1^.

### Reporting summary

Further information on research design is available in the Nature Portfolio Reporting Summary linked to this article.

## Supplementary information

Supplementary Information

Reporting Summary

Description of Additional Supplementary Files

Supplementary Datasets 1–4

## Data Availability

Source data are provided with this paper. The δ^18^O_b_ data and coulometric CaCO_3_ content generated in this study and the depth-age tie points used in the astronomical tuning have also been archived in the Pangaea database^79^ (10.1594/PANGAEA.958176).

## Code availability

The code used in this study are available in the Github repository^80^ (https://github.com/timvanpeer/IODP_U1406).
