# Supplementary material for: Eccentricity pacing and rapid termination of the early Antarctic ice ages
Source: Nat Commun. 2024 Dec 5;15:10600. doi: 10.1038/s41467-024-54186-1 (PMC11621355; doi:10.1038/s41467-024-54186-1)
Supplement: Supplementary file 1 — Supplementary Information [file 41467_2024_54186_MOESM1_ESM.pdf]

Supplementary Information

of "Eccentricity pacing and rapid termination of the early Antarctic ice ages"

Tim E. van Peer[1,2,3,*], Diederik Liebrand[1,4], Victoria E. Taylor[1,5], Swaantje Brzelinski[6], Iris Wolf[7], André Bornemann[8], Oliver Friedrich[6], Steven M. Bohaty[6], Chuang Xuan[1], Peter C. Lippert[9], and Paul A. Wilson[1]

[1]University of Southampton, Waterfront Campus, National Oceanography Centre Southampton, UK.

[2]Department of Earth Sciences, University College London, London, UK.

[3]School of Geography, Geology, and the Environment, University of Leicester, Leicester, UK.

[4]Department of Earth and Environmental Sciences, The University of Manchester, Manchester, UK.

[5]Department of Earth Science, Bjerknes Centre for Climate Research, University of Bergen, Bergen, Norway.

[6]Institute of Earth Sciences, Ruprecht-Karls-Universität Heidelberg, Heidelberg, Germany.

[7]Institute of Geosciences, Goethe-University Frankfurt, Frankfurt, Germany.

[8]Federal Institute for Geosciences and Natural Resources, Hannover, Germany.

[9]Department of Geology & Geophysics, University of Utah, Salt Lake City, USA.

***** t.e.vanpeer@leicester.ac.uk

Astronomical tuning

Cyclostratigraphy

The $CaCO_3$ content of the Site U1406 study interval oscillates with a period of ~1 m between 165–145 m and 95–35 m CCSF-M; this cyclicity is also observed in the composite core images (Fig. S6a). Wavelet analysis of $CaCO_3$ content supports these observations: clear cyclicity with a wavelength of ~1.2 m (0.86 cycles m^{-1}) is present between 165–145 m and 95–35 m CCSF-M (Fig. S7e).

Magnetostratigraphic reversal boundaries were identified through high-resolution shore-based palaeomagnetic analysis [1]. Chrons C9n to C6Ar (~27–21 Ma; Fig. S7a) were identified in the Site U1406 study interval, facilitating initial estimates of sedimentation rates using the GTS2012 reversal ages [2,3]. The observed sedimentation rates of ~3 cm kyr^{-1} in Intervals III and I yield a duration of ~40 kyr for the dominant ~1.2-m cycles, indicating that these thinner cycles are likely paced by obliquity.

Astrochronology

To derive the first-order phase relationship between $CaCO_3$ content and obliquity we: 1) evaluated the phase relationship between $CaCO_3$ content and $\delta^{18}O_b$ on the magnetostratigraphic age model and 2) assumed the phase relationship of $\delta^{18}O_b$ to obliquity is the same as that during the Pleistocene [4], where maxima in $\delta^{18}O_b$ coincide with obliquity minima. Maxima in $\delta^{18}O_b$ lag minima in $CaCO_3$ by ~7 kyr (Fig. S5b). We, therefore, correlate minima in $CaCO_3$ to minima in Earth's obliquity solution. For consistency with previous studies [5–7] and because they are not constrained by geological data beyond ~10 Ma [8], we used the La2004 solution as target curve with present-day values for tidal dissipation and dynamical ellipticity (i.e., we do not attempt to make corrections for changes in the duration of obliquity cycles caused by effects of tidal dissipation and dynamical ellipticity [9]). We obtained the final fine-tuned ages by aligning minima of individual ~1.2 m cycles of $CaCO_3$ content to minima in the 40-kyr obliquity cycles (Dataset S3). Continuous tuning is achieved for most of the $CaCO_3$ record (Fig. S7g), constraining it to ~27.1–21.0 Ma (Fig. S7f–S7i), within the Chattian to Aquitanian stages and 405-kyr Eccentricity Cycles 67 to 53. Our tuning dates our $\delta^{18}O_b$ record to span between ~26.2 and 21.8 Ma.

We validated the accuracy of the $CaCO_3$-obliquity tuned age model by comparing the eccentricity filters of the $\delta^{18}O_b$ data with the same filters of the La2004 eccentricity solution [9], both for the short (~110-kyr) and long (405-kyr) eccentricity cycles (Fig. S8c,d). Both filters are in good agreement throughout the record. A second, independent validation method compared the 173-kyr amplitude modulation of a broad obliquity filter of $CaCO_3$ with that to similar computations based on the

La2004 obliquity solution [9,10]. The result again shows a good agreement between the two amplitude modulations (Fig. S8e).

Data quality control

Selected intervals from sites 926, 1090, and 1218 were omitted from the variance calculations because the data series lacked temporal resolution (here defined as 200 kyr-long intervals, with fewer than an average of 4 data points per obliquity cycle [11]). Using this criterion, we omitted early Miocene data from ODP Site 926 [6] between 20.2–19.7 Ma, early Miocene data from ODP Site 1090 [7] between 22.2–21 Ma, and latest Oligocene data from ODP Site 1218 [5] between 23.4–22.2 Ma (Supplementary Data 3).

The $^{18}O_b$ record from Site 1090 is from a mix of *Oridorsalis umbonatus* and *Cibicidoides* species [7]. The record from Site 1218 was also generated on various *Cibicidoides* species [5]. At Site 926, the $\delta^{18}O_b$ record was produced using a mixture of *Cibicidoides* species in the interval between ~26.5–25 Ma (Ref. [6]) and uses only *Cibicidoides mundulus* between 25.0–17.9 Ma. The $\delta^{18}O_b$ record from Site 1264 is derived from *Cibicidoides mundulus* only and sampled at high resolution, but it is possible that the obliquity-paced signal is damped or even removed by sediment mixing (bioturbation), because sedimentation rates are low (often ≤ 1cm kyr^{-1}, Fig. 3b, d, S2g), In contrast, all other studied sites have sedimentation rates >1 cm kyr^{-1} (Supplementary Data 3).

We note the absolute offset between the $\delta^{18}O_b$ record of Site 926 relative to that of all other studied sites. This offset has been observed elsewhere [12] as part of evaluation of isotopic gradients (including $\delta^{13}C_b$) Sites 926, 1090, 1218, and 1264. We hypothesise this 0.5-‰ offset in $\delta^{18}O_b$ (equivalent to *ca.* 2 °C) is due to diagenesis at depth. With uncertainties in geothermal gradient of 0.03-0.047 °C m^{-1} (Refs. [13,14]), this equates to 10−15% local diagenesis.

In summary, the $\delta^{18}O_b$ record at Site U1406 is the only record that passes all four quality criteria (completeness, sedimentation rate, foraminifera, preservation, see Supplementary Data 3), with the other records affected by low sedimentation rates (Site 1264), insufficient sampling (Sites 926, 1090,

1218), generated using multiple genera or species (Sites 1090 and 1218, and the older ~1.5 Myr of Site 926), and/or preservation or diagenetic artefacts (Sites 926 and 1090).

We also evaluated the potential effect of tuning obliquity and/or eccentricity into a record, artificially enhancing or reducing V_{Obl} and/or V_{Ecc} values. We calculated V_{Obl} and V_{Ecc} of our $\delta^{18}O_b$ record using three different independent age models, i.e., our tuning to obliquity, using GTS2012 reversal ages [2,3], and using reversal ages calculated from spreading rates with a revised age for the Oligocene-Miocene Boundary reversal [15,16]. Despite large absolute age differences up to 150 kyr, these different age models do not yield substantially different V_{Obl} and V_{Ecc} (Fig. S9).

Age model changes at Site 1218 were also evaluated to establish any influence on the V_{Obl} and V_{Ecc} patterns. The $\delta^{18}O_b$ record of Site 1218 was originally tuned to an ETP curve [5], which was later re-tuned solely to eccentricity [17]. This revised tuning yields large differences in the absolute ages of some $\delta^{18}O_b$ data points (Fig. S10a), but the corresponding V_{Ecc} or V_{Obl} values are identical for the majority of the record (Fig. S10b, 10c). We observe that data processing (e.g., de-trending, interpolation, removal of outliers) may yield minor offsets, demonstrated by a comparison between the V_{Obl} and V_{Ecc} computed from the $\delta^{18}O_b$ megasplice [18] and the original records of sites 1218 (28–24.5 Ma), 1090 (*ca.* 23 Ma), and 926 (22-21 Ma), but these offsets are trivial and cannot explain the observed site-to-site discrepancies in V_{Obl}.

Supplementary figures

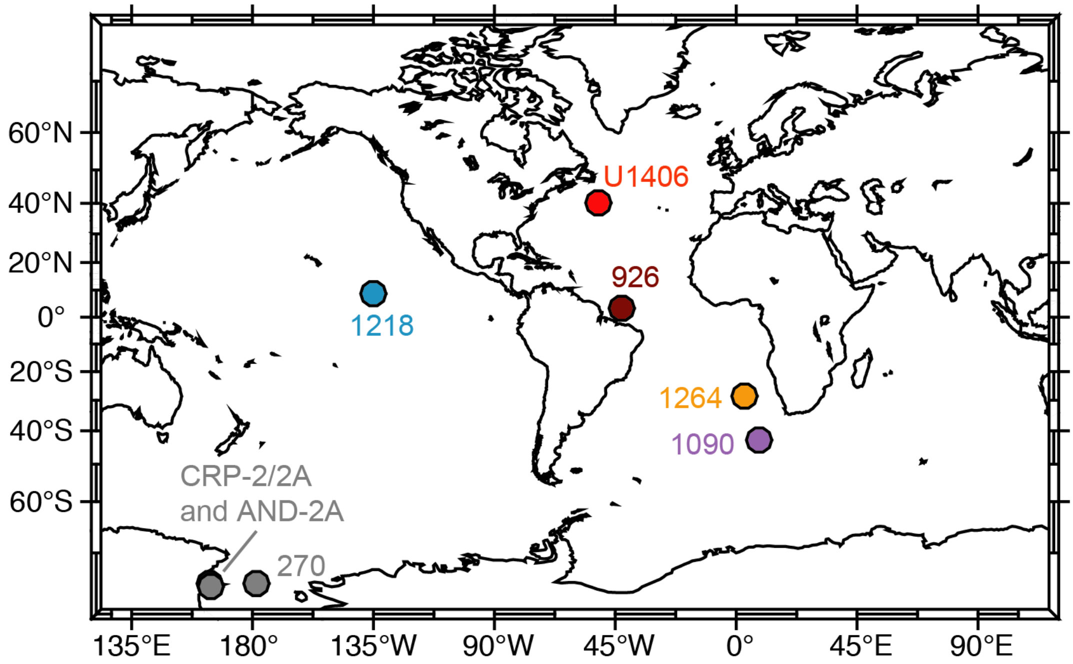

Fig. S1. Location of Integrated Ocean Drilling Program Site U1406 (bright red) and other site localities discussed in this study are also shown including sites 926 (dark red), 1090 (purple), 1218 (blue), 1264 (yellow), and ice-proximal localities Site 270, ANDRILL (AND) 2A, and Cape Roberts Project (CRP) Site 2/2A (grey). Note the close proximity of AND-2A and CRP-2/2A so their site symbols are plotted on top of each other.

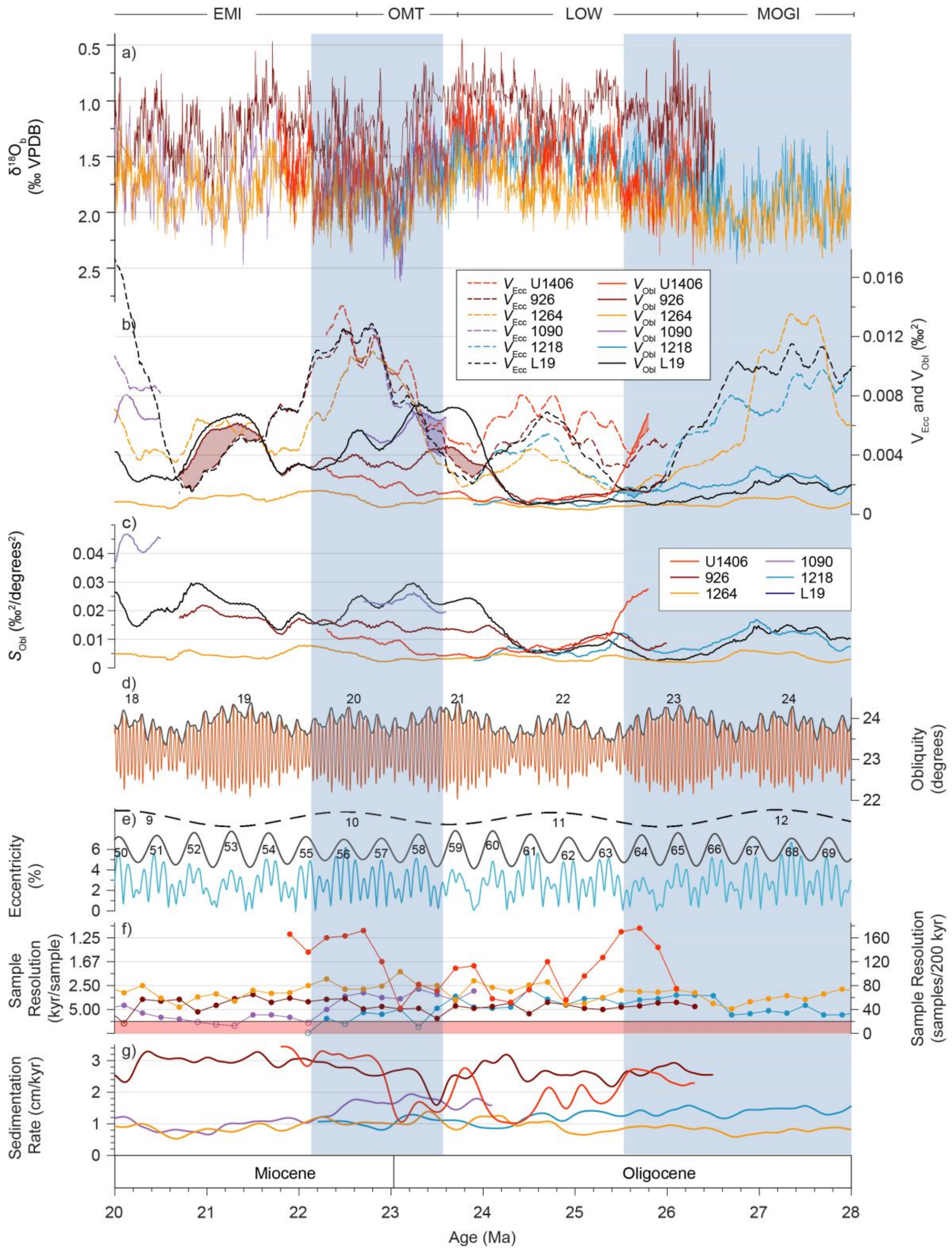

Fig. S2. As for Fig. 3 but with further information on the fidelity of the records. a) $\delta^{18}O_b$ records of ODP/IODP sites U1406 (bright red; presented here), 926 (dark red) [6], 1090 (purple) [7], 1218 (blue) [5], and 1264 (yellow) [19] during EMI (Early Miocene Interval), OMT (Oligocene-Miocene Transition),

LOW (Late Oligocene Warming), and MOGI (Mid-Oligocene Glacial Interval). Blue shading indicates colder intervals (roughly equivalent to the MOGI/LOW transition and OMT interval) with high-amplitude variability in $\delta^{18}O_b$ on frequencies corresponding to eccentricity. b) V_{Obl} (continuous lines) and V_{Ecc} (dashed lines) of the $\delta^{18}O_b$ records shown in a) and the composite $\delta^{18}O_b$ record (L19) [18,20]. Shaded intervals highlight where $V_{Obl} > V_{Ecc}$ in individual records. c) Obliquity sensitivity (S_{Obl}) [20] represents $\delta^{18}O_b$ response to astronomical forcing and is defined by the ratio between V_{Obl} in $\delta^{18}O_b$ and V_{Obl} in the La2004 obliquity solution [9] (see Site-to-site variability in the response of $\delta^{18}O_b$ to obliquity). d) and e) The La2004 obliquity and eccentricity solutions [9], respectively. Long-term eccentricity modulations of ~405 kyr and 2.4 Myr are offset in e) for visibility. f) temporal resolution of these five $\delta^{18}O_b$ records. Open dots and red shading indicate insufficient data resolution to resolve obliquity. Note the exceptional resolution of Site U1406 presented here. g) smoothed sedimentation rates at the five sites.

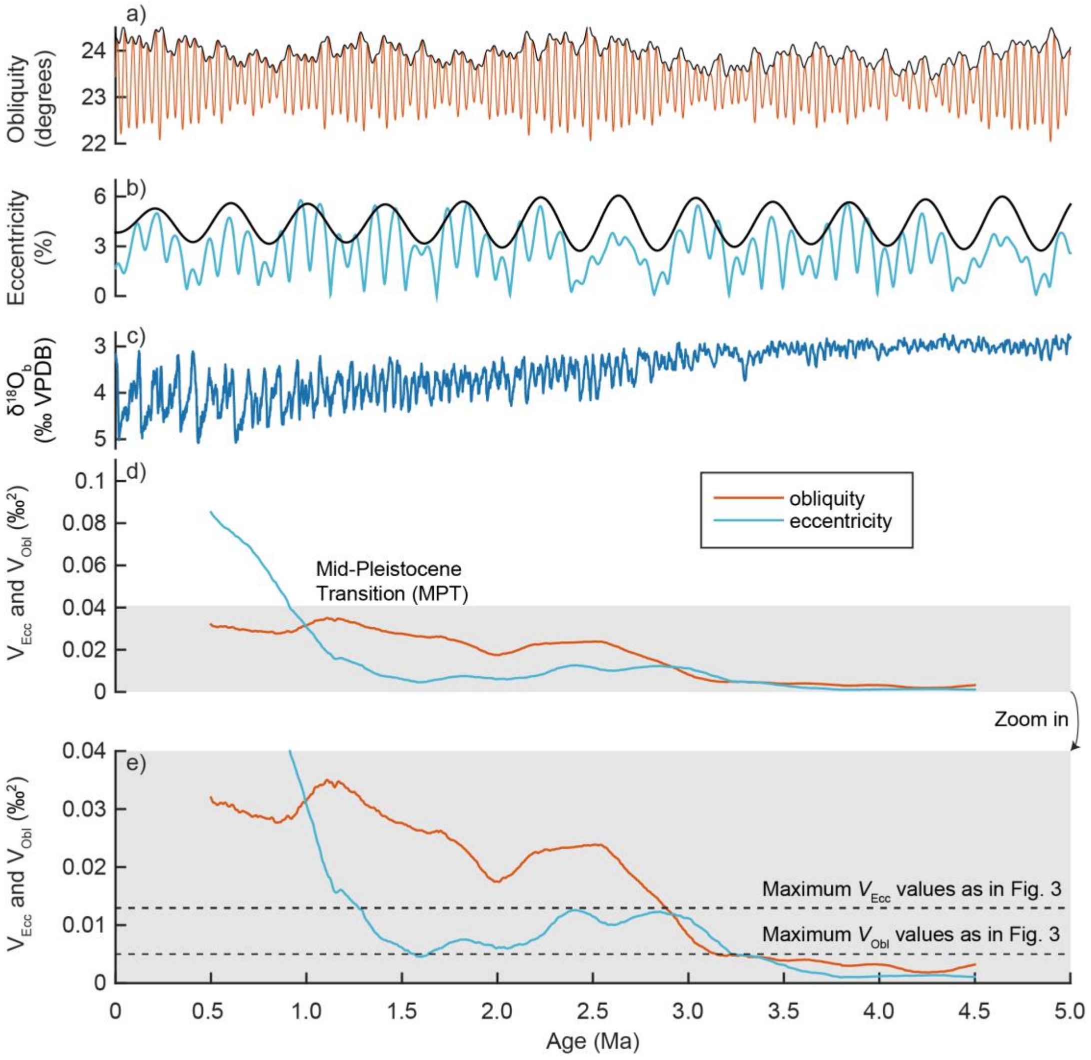

Fig. S3. The response to eccentricity and obliquity in the global $\delta^{18}O_b$ stack over the last 5 Myr. a) and b) Astronomical parameters obliquity and eccentricity, respectively. c) The $\delta^{18}O_b$ stack of LR04 [21] from which we compute V_{Ecc} and V_{Obl} in d) and e). e) is zoomed in on lower half of d) to emphasise variations during the Pliocene and prior to the Mid-Pleistocene Transition.

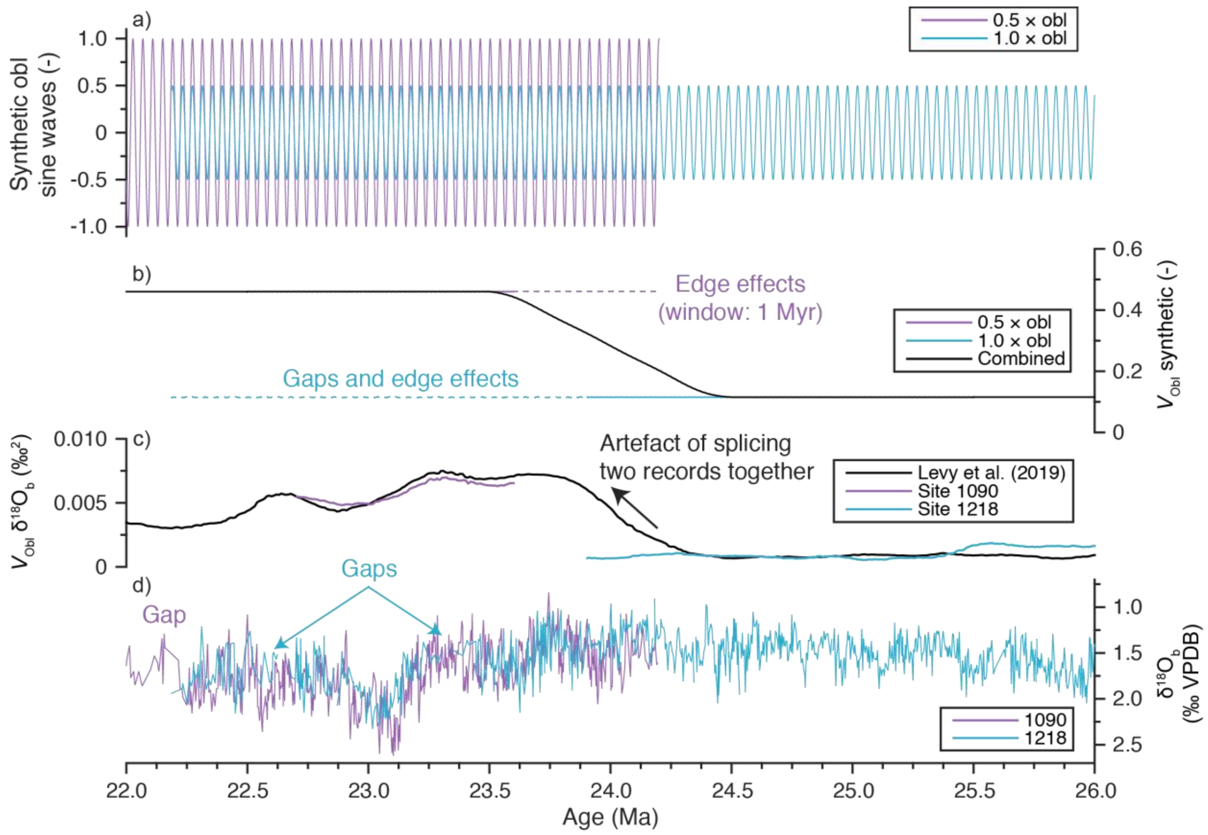

Fig. S4. Synthetic representation of the distortion caused by splicing two records together. a) two obliquity-like sine waves with amplitude 0.5 (blue) and 1.0 (purple) from the synthetic V_{Obl} in b). Note that the transition from the sine wave with amplitude 0.5 to the sine wave with amplitude 1.0 is smeared out over the length of the window used (here 1 Myr, see Methods). This closely resembles the observed V_{Obl} pattern at 24 Ma from Ref. [20] in c), splicing together the $\delta^{18}O_b$ records of Sites 1218 and 1090 visible in d).

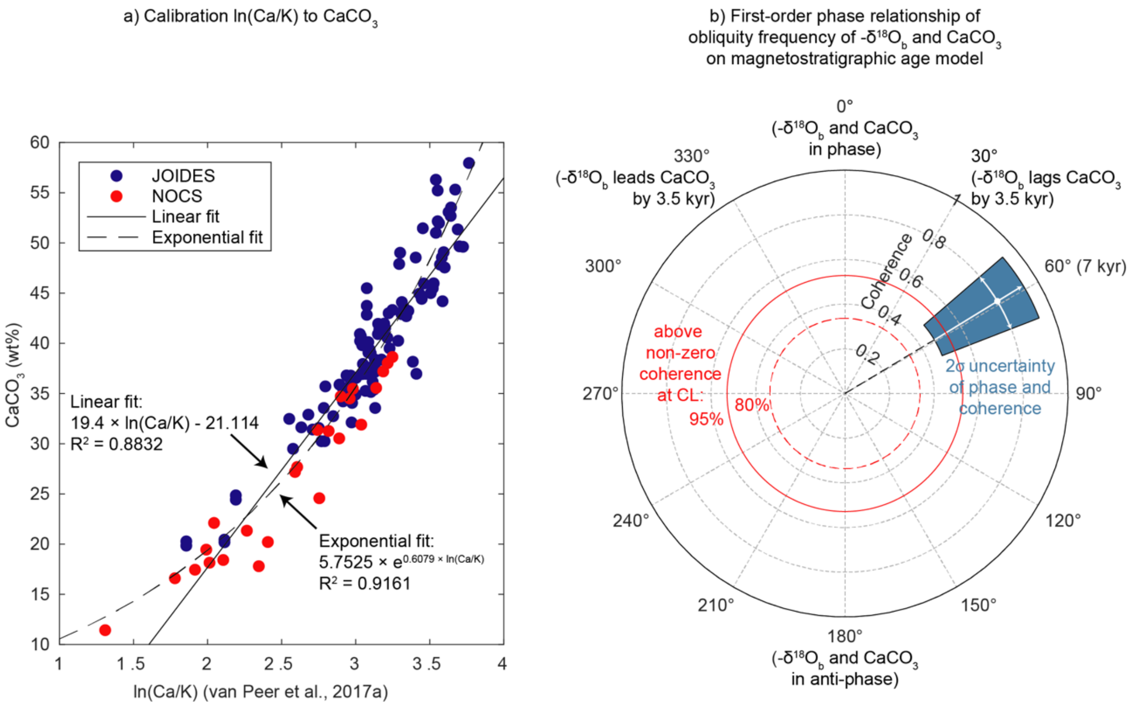

Fig S5. X-ray fluorescence (XRF) calibration and initial phase analysis. a) XRF ln(Ca/K) data [22] plotted against shipboard [23] and newly generated coulometric $CaCO_3$ content from the same depths. b) Phase relationship between $CaCO_3$ content and $\delta^{18}O$, highlighting maxima in $\delta^{18}O$ lag minima in $CaCO_3$ by ~7 kyr. Blue shaded interval indicates 2σ uncertainties in phase and coherence. Red circles denote when $CaCO_3$ and $\delta^{18}O$ are coherent at 80% (dashed) and 95% (continuous) significance levels (CL).

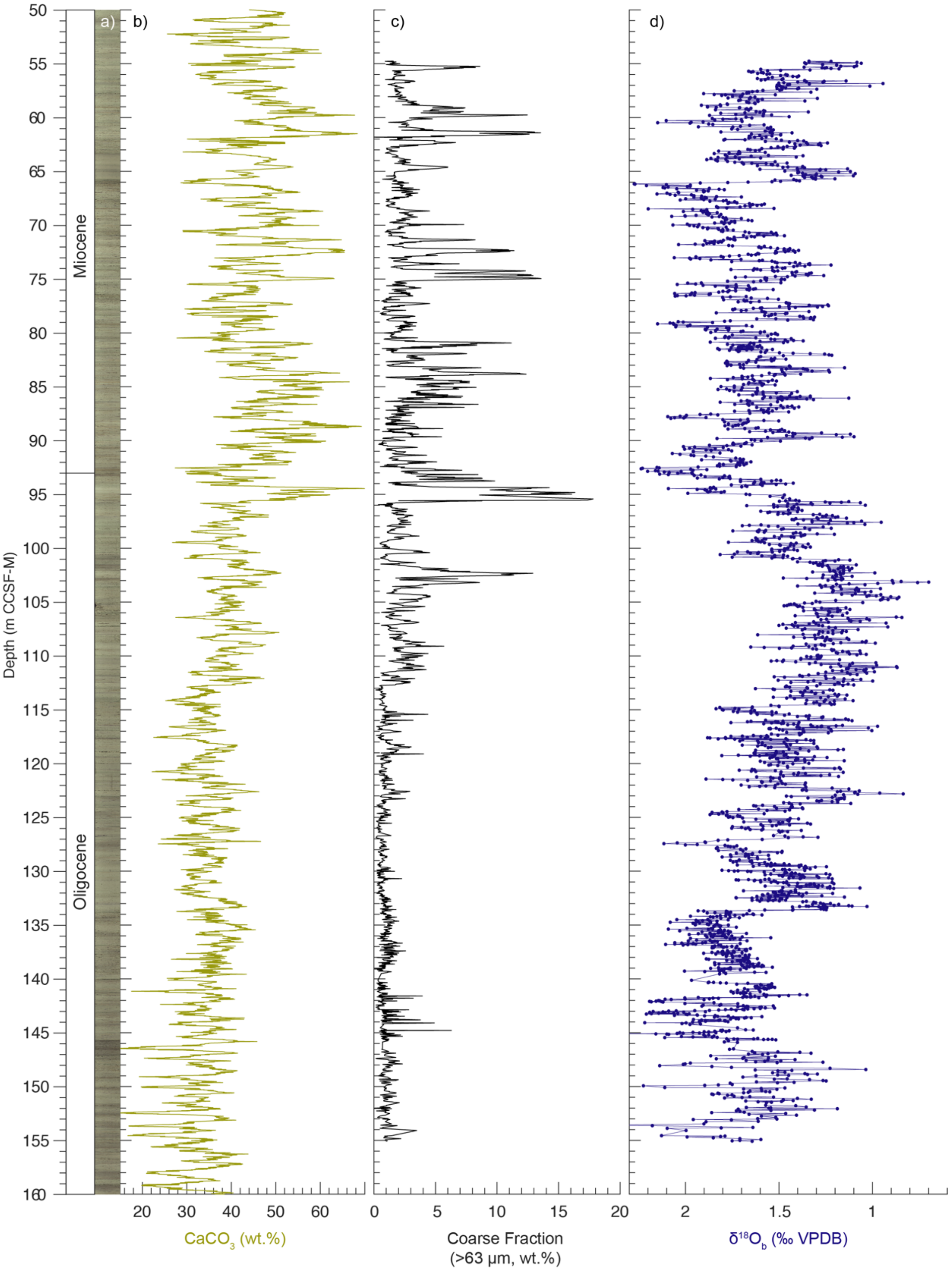

Fig. S6. Our new record from IODP Site U1406 in the depth domain. a) Spliced linescan images of studied core sections of IODP Site U1406 [22], b) $CaCO_3$ content, c) coarse fraction (>63 µm), and d) benthic foraminiferal stable oxygen ($\delta^{18}O_b$) isotope ratios.

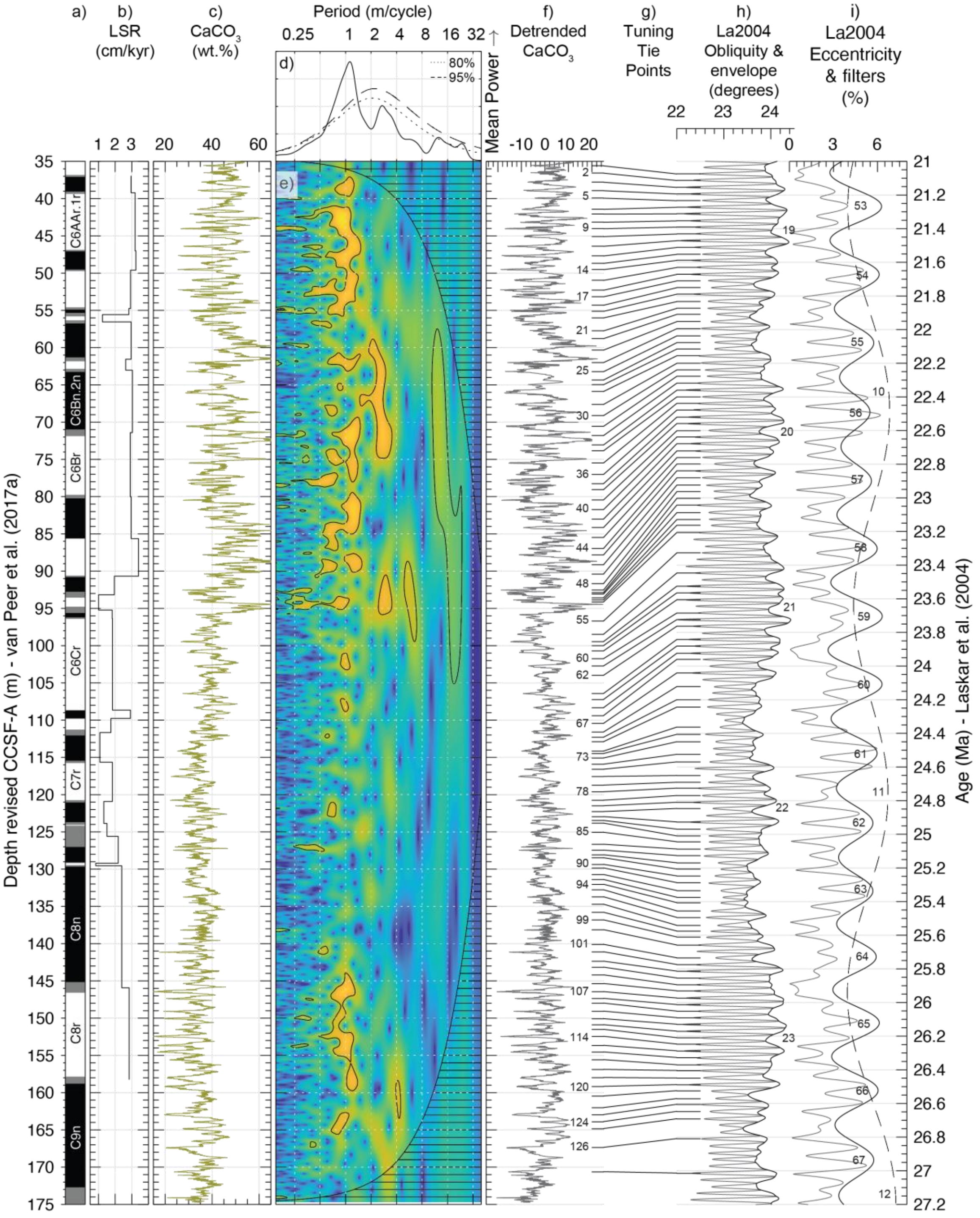

Fig. S7. Cyclostratigraphic analysis in the depth domain and tuning to the full obliquity curve. a) and b) Magnetostratigraphy and sedimentation rates [1], respectively, based on reversal ages from GTS2012 [2,3]. c). $CaCO_3$ content. d) Global and e) local wavelet power spectra of detrended (periodicities >25 m removed) $CaCO_3$ content. Dotted and dashed lines in d) denote 80% and 95% significance levels, respectively. f) detrended $CaCO_3$ to highlight numbered cycles used in astronomical tuning presented in g) between $CaCO_3$ content data and h) the obliquity orbital solution from La2004 [9], including

amplitude modulation. i) For comparison, the La2004 eccentricity solution [9]. The 405-kyr and ~2.4-Myr eccentricity band-pass filtered components are offset to aid visibility.

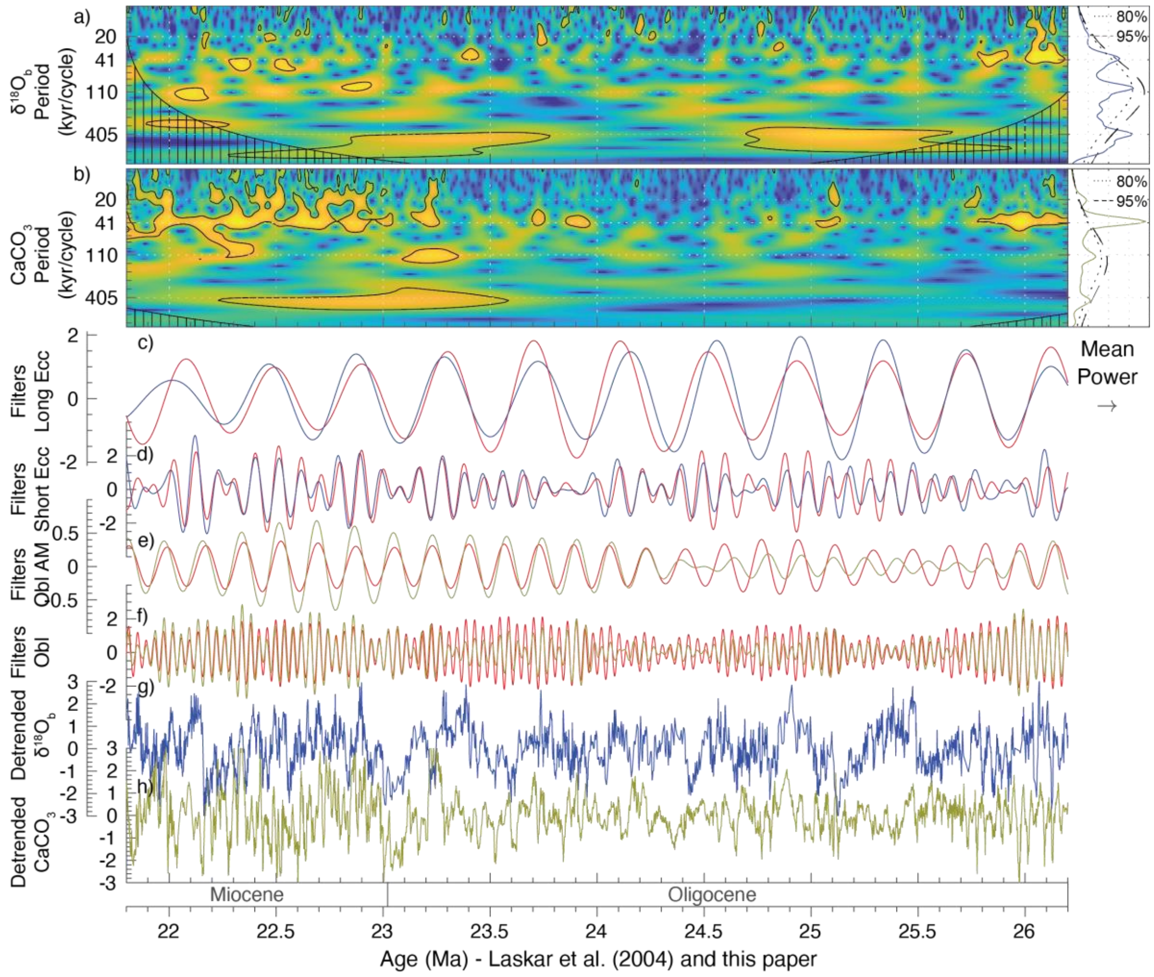

Fig. S8. Cyclostratigraphic analysis in the age domain. a) and b) show the local and global wavelet power spectra of detrended (periodicities >1 Myr removed) $CaCO_3$ content and $\delta^{18}O_b$. c) and d) Filtered $\delta^{18}O_b$ (blue), and La2004 eccentricity (red) [9] using band-pass filters equivalent to the long (2.5 ± 0.5 cycles Myr^{-1}) and short (9 ± 3 cycles Myr^{-1}) eccentricity (Ecc) cycles. e) amplitude modulation (AM) computed on the filters of $CaCO_3$ content (yellow) and La2004 obliquity solution [9] (bandwidth 23 ± 5 cycles Myr^{-1}) presented in f). g) and h) $CaCO_3$ content (yellow) and $\delta^{18}O$ (blue) records before cyclostratigraphic analysis, after detrending (see Methods). Note the excellent agreement between the La2004 eccentricity [9] and $\delta^{18}O_b$ eccentricity-bandwidth filter (blue and red in d), respectively), even though the tuning assumption is solely $CaCO_3$ content to the La2004 obliquity curve.

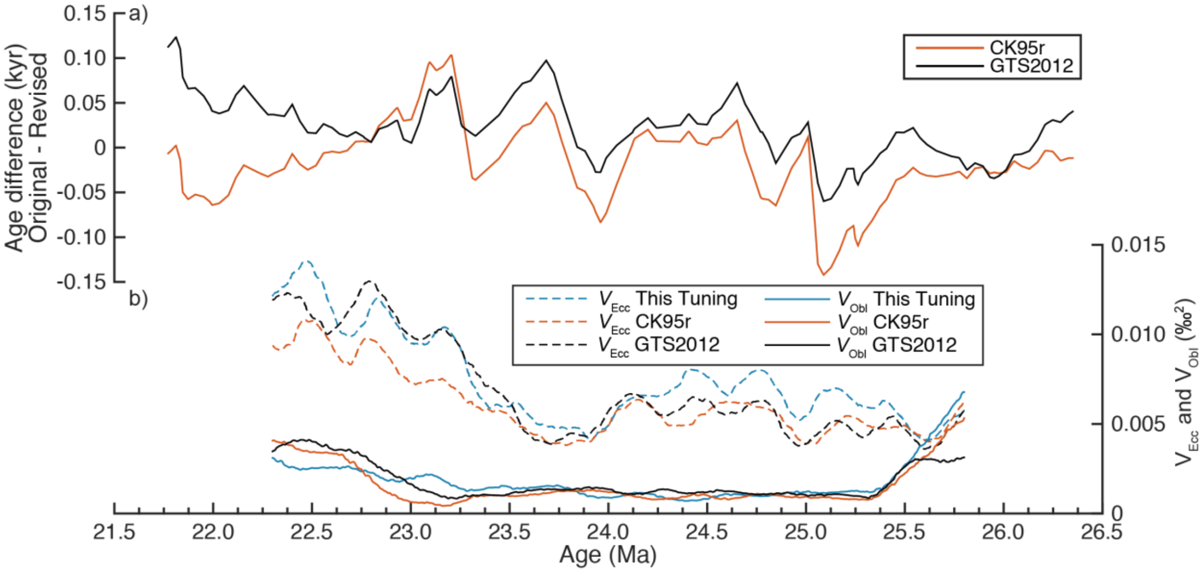

Fig. S9. Effect of age model uncertainties on V_{Obl} and V_{Ecc} at Site U1406. a) differences in absolute age between the astronomically tuned age model presented here and that based on magnetostratigraphic tie points[1] correlated to two widely used age model options GTS2012[2,3] and CK95r[15] with an updated age tie point at the Oligocene-Miocene Boundary[16]. The V_{Obl} and V_{Ecc} from each of these age models show some subtle differences due to the exact age tie points used but the overarching patterns are practically identical.

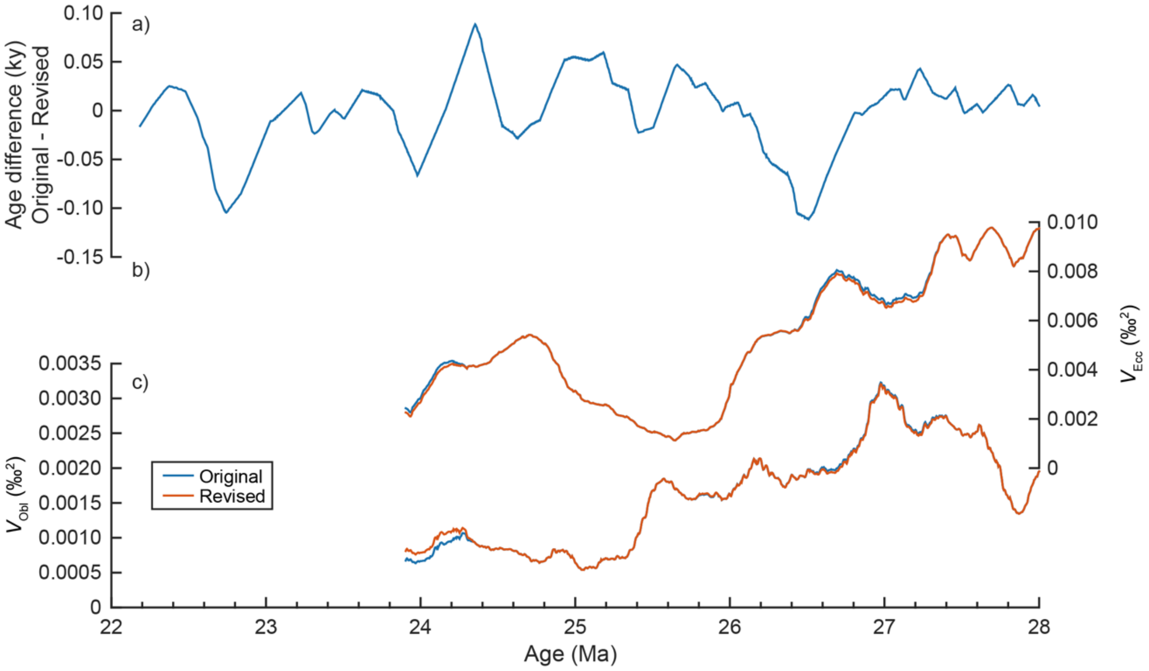

Fig. S10. Effect of age model updates on V_{Obl} and V_{Ecc} at ODP Site 1218. a) differences in absolute age of individual data points between the original age model [5] and a revised version [17], with b) and c) corresponding V_{Ecc} and V_{Obl} values, respectively.

Supplementary References

1. van Peer, T. E. *et al.* Extracting a Detailed Magnetostratigraphy From Weakly Magnetized, Oligocene to Early Miocene Sediment Drifts Recovered at IODP Site U1406 (Newfoundland Margin, Northwest Atlantic Ocean). *Geochemistry, Geophysics, Geosystems* **18**, 3910–3928 (2017).
2. Hilgen, F. J., Lourens, L. J. & Van Dam, J. A. The Neogene Period. in *The Geological Time Scale* (eds. Gradstein, F. M., Ogg, J. G., Schmitz, M. D. & Ogg, G. M.) 923–978 (Elsevier B.V., Amsterdam, 2012).
3. Vandenberghe, N., Hilgen, F. J. & Speijer, R. P. The Paleogene Period. in *A Geological Time Scale* (eds. Gradstein, F. M., Ogg, J. G., Schmitz, M. & Ogg, G.) 855–921 (Elsevier B.V., Amsterdam, 2012). doi:DOI:10.1016/B978-0-444-59425-9.00028-7.
4. Hays, J. D., Imbrie, J. & Shackleton, N. J. Variations in the Earth's Orbit: Pacemaker of the Ice Ages. *Science* **194**, 1121–1132 (1976).
5. Pälike, H. *et al.* The Heartbeat of the Oligocene Climate System. *Science (1979)* **314**, 1894–1898 (2006).
6. Pälike, H., Frazier, J. & Zachos, J. C. Extended orbitally forced palaeoclimatic records from the equatorial Atlantic Ceara Rise. *Quat Sci Rev* **25**, 3138–3149 (2006).
7. Billups, K., Pälike, H., Channell, J. E. T., Zachos, J. C. & Shackleton, N. J. Astronomic calibration of the late Oligocene through early Miocene geomagnetic polarity time scale. *Earth Planet Sci Lett* **224**, 33–44 (2004).
8. Zeeden, C., Hilgen, F. J., Hüsing, S. K. & Lourens, L. L. The Miocene astronomical time scale 9-12-Ma: New constraints on tidal dissipation and their implications for paleoclimatic investigations. *Paleoceanography* **29**, 296–307 (2014).
9. Laskar, J. *et al.* A long-term numerical solution for the insolation quantities of the Earth. *Astron Astrophys* **285**, 261–285 (2004).
10. Zeeden, C., Meyers, S. R., Hilgen, F. J., Lourens, L. J. & Laskar, J. Time scale evaluation and the quantification of obliquity forcing. *Quaternary Science Reviews* **209**, 100–113 (2019).
11. Martinez, M., Kotov, S., De Vleeschouwer, D., Pas, D. & Pälike, H. Testing the impact of stratigraphic uncertainty on spectral analyses of sedimentary series. *Climate of the Past* **12**, 1765–1783 (2016).
12. Beddow, H. M., Liebrand, D., Sluijs, A., Wade, B. S. & Lourens, L. J. Global change across the Oligocene-Miocene Transition: High-resolution stable isotope records from IODP Site U1334 (equatorial Pacific Ocean). *Paleoceanography* **31**, 81–97 (2016).
13. Shipboard Scientific Party. Site 926. in *Proceedings of the Ocean Drilling Program, Initial Reports* (eds. Curry, W. B., Shackleton, N. J., Richter, C. & Al., E.) vol. 154 153–232 (Ocean Drilling Program, College Station, TX, 1995).
14. Parsons, B. & Sclater, J. G. An Analysis of the Variation of Ocean Floor Bathymetry and Heat Flow with Age. *Journal of Geophysical Research* **82**, 803–827 (1977).
15. Cande, S. C. & Kent, D. V. Revised calibration of the geomagnetic polarity timescale for the Late Cretaceous and Cenozoic. *Journal of Geophysical Research* **100**, 6093–6095 (1995).

16. Shackleton, N. J., Hall, M. A., Raffi, I., Tauxe, L. & Zachos, J. Astronomical calibration age for the Oligocene-Miocene boundary. *Geology* **28**, 447–450 (2000).

17. Westerhold, T. *et al.* An astronomically dated record of Earth’s climate and its predictability over the last 66 million years. *Science (1979)* **369**, 1383–1388 (2020).

18. De Vleeschouwer, D., Vahlenkamp, M., Crucifix, M. & Pälike, H. Alternating Southern and Northern Hemisphere climate response to astronomical forcing during the past 35 m.y. *Geology* **45**, 375–378 (2017).

19. Liebrand, D. *et al.* Evolution of the early Antarctic ice ages. *PNAS* **114**, 3867–3872 (2017).

20. Levy, R. H. *et al.* Antarctic ice-sheet sensitivity to obliquity forcing enhanced through ocean connections. *Nat Geosci* **12**, 132–137 (2019).

21. Lisiecki, L. E. & Raymo, M. E. A Pliocene-Pleistocene stack of 57 globally distributed benthic D 18 O records. *Paleoceanography* **20**, PA1003 (2005).

22. van Peer, T. E. *et al.* Data report: revised composite depth scale and splice for IODP Site U1406. in *Proceedings of the Integrated Ocean Drilling Program* (eds. Norris, R. D., Wilson, P. A., Blum, P. & Scientists Expedition 342) vol. 342 1–23 (Integrated Ocean Drilling Program, College Station, TX, 2017).

23. Norris, R. D. *et al.* Site U1406. in *Proceedings of the Integrated Ocean Drilling Program* (eds. Norris, R. D. et al.) vol. 342 (Integrated Ocean Drilling Program, College Station, TX, 2014).
