# Supplementary material for: Eccentricity pacing and rapid termination of the early Antarctic ice ages
Source: Nat Commun. 2024 Dec 5;15:10600. doi: 10.1038/s41467-024-54186-1 (PMC11621355; doi:10.1038/s41467-024-54186-1)
Supplement: Supplementary file 3 — Description of Additional Supplementary Files [file 41467_2024_54186_MOESM3_ESM.pdf]

Description of Additional Supplementary Files:

Supplementary Data 1: Coarse fraction and $\delta^{18}O_b$ data generated in this study.

Supplementary Data 2: Depth-to-age tie points used in the astronomical tuning.

Supplementary Data 3: Quality criteria used to analyse the $\delta^{18}O_b$ records of Sites 926, 1090, 1218, 1264, and U1406 (presented here). Records are considered complete when there are no gaps, here defined as insufficient sampling resolution (>10 kyr sample^{-1}) over prolonged intervals (>200 kyr).

Supplementary Data 4: Coulometric carbonate content data generated in this study
